# The impact of mosquito sampling strategies on molecular xenomonitoring prevalence for filariasis: a systematic review

**DOI:** 10.2471/BLT.23.290424

**Published:** 2023-12-08

**Authors:** Lisa J Reimer, Joseph D Pryce

**Affiliations:** a Department of Vector Biology, Liverpool School of Tropical Medicine, Pembroke Place, Liverpool, L3 5QA, England.

## Abstract

**Objective:**

To explore the impact of mosquito collection methods, sampling intensity and target genus on molecular xenomonitoring detection of parasites causing lymphatic filariasis.

**Methods:**

We systematically searched five databases for studies that used two or more collection strategies for sampling wild mosquitoes, and employed molecular methods to assess the molecular xenomonitoring prevalence of parasites responsible for lymphatic filariasis. We performed generic inverse variance meta-analyses and explored sources of heterogeneity using subgroup analyses. We assessed methodological quality and certainty of evidence.

**Findings:**

We identified 25 eligible studies, with 172 083 mosquitoes analysed. We observed significantly higher molecular xenomonitoring prevalence with collection methods that target bloodfed mosquitoes compared to methods that target unfed mosquitoes (prevalence ratio: 3.53; 95% confidence interval, CI: 1.52–8.24), but no significant difference compared with gravid collection methods (prevalence ratio: 1.54; 95% CI: 0.46–5.16). Regarding genus, we observed significantly higher molecular xenomonitoring prevalence for anopheline mosquitoes compared to culicine mosquitoes in areas where *Anopheles* species are the primary vector (prevalence ratio: 6.91; 95% CI: 1.73–27.52). One study provided evidence that reducing the number of sampling sites did not significantly affect molecular xenomonitoring prevalence. Evidence of differences in molecular xenomonitoring prevalence between sampling strategies was considered to be of low certainty, due partly to inherent limitations of observational studies that were not explicitly designed for these comparisons.

**Conclusion:**

The choice of sampling strategy can significantly affect molecular xenomonitoring results. Further research is needed to inform the optimum strategy in light of logistical constraints and epidemiological contexts.

## Résumé

**Objectif:**

Explorer l’impact des méthodes de collecte de moustiques, de l’intensité de l’échantillonnage et du genre cible sur la détection par xénosurveillance moléculaire des parasites responsables de la filariose lymphatique.

**Méthodes:**

Nous avons recherché systématiquement, dans cinq bases de données, des études recourant à au moins deux stratégies de collecte en vue de l’échantillonnage de moustiques à l’état sauvage et appliquant des méthodes moléculaires pour évaluer la prévalence des parasites responsables de la filariose lymphatique dans le cadre d’une xénosurveillance moléculaire. Nous avons effectué des méta-analyses génériques de variance inverse et exploré les sources d’hétérogénéité à l’aide d’analyses de sous-groupes. Nous avons évalué la qualité méthodologique et la certitude des preuves.

**Résultats:**

Nous avons identifié 25 études admissibles, avec 172 083 moustiques analysés. Nous avons observé une prévalence de xénosurveillance moléculaire significativement plus élevée avec les méthodes de collecte ciblant les moustiques hématophages qu’avec les méthodes ciblant les moustiques non hématophages (rapport de prévalence: 3,53; intervalle de confiance (IC) à 95%: 1,52–8,24), mais aucune différence significative par rapport aux méthodes de collecte de femelles gravides (rapport de prévalence: 1,54; IC à 95%: 0,46–5,16). En ce qui concerne le genre, nous avons observé une prévalence de xénosurveillance moléculaire significativement plus élevée pour les moustiques anophèles que pour les moustiques culicidés dans les zones où les espèces d’anophèles sont le vecteur principal (rapport de prévalence: 6,91; IC à 95%: 1,73–27,52). Une étude a mis en évidence que la réduction du nombre de sites d’échantillonnage n’avait pas d’incidence significative sur la prévalence de la xénosurveillance moléculaire. Les preuves de différences dans la prévalence de la xénosurveillance moléculaire entre les stratégies d’échantillonnage ont été considérées comme peu sûres, en partie à cause des limites inhérentes aux études d’observation qui n’ont pas été explicitement conçues pour ces comparaisons.

**Conclusion:**

Le choix de la stratégie d’échantillonnage peut affecter de manière significative les résultats de la xénosurveillance moléculaire. Des recherches supplémentaires sont nécessaires pour déterminer la stratégie optimale en fonction des contraintes logistiques et des contextes épidémiologiques.

## Resumen

**Objetivo:**

Explorar el impacto de los métodos de recolección de mosquitos, la intensidad del muestreo y el género objetivo en la detección mediante xenomonitoreo molecular de los parásitos que causan la filariasis linfática.

**Métodos:**

Se realizaron búsquedas sistemáticas en cinco bases de datos de estudios que utilizaron dos o más estrategias de recolección para el muestreo de mosquitos silvestres y que emplearon métodos moleculares para evaluar la prevalencia de xenomonitoreo molecular de los parásitos responsables de la filariasis linfática. Se realizaron metanálisis genéricos de varianza inversa y se exploraron las fuentes de heterogeneidad mediante análisis de subgrupos. Se evaluó la calidad de la metodología y la certeza de las evidencias.

**Resultados:**

Se identificaron 25 estudios elegibles, con 172 083 mosquitos analizados. Se observó una prevalencia significativamente mayor de xenomonitoreo molecular con métodos de recolección dirigidos a mosquitos hematófagos en comparación con métodos dirigidos a mosquitos no hematófagos (razón de prevalencia: 3,53; intervalo de confianza del 95%, IC: 1,52-8,24), pero ninguna diferencia significativa en comparación con los métodos de recolección de hembras gestantes (razón de prevalencia: 1,54; IC del 95%: 0,46-5,16). En cuanto al género, se observó una prevalencia significativamente mayor de xenomonitoreo molecular para los mosquitos anofelinos en comparación con los culícidos en zonas donde las especies de *Anopheles* son el vector principal (razón de prevalencia: 6,91; IC del 95%: 1,73-27,52). Un estudio aportó evidencias de que la reducción de la cantidad de sitios de muestreo no afectó significativamente la prevalencia del xenomonitoreo molecular. Las evidencias de diferencias en la prevalencia del xenomonitoreo molecular entre las estrategias de muestreo se consideraron de baja certeza, lo que se debió en parte a las limitaciones inherentes de los estudios observacionales que no se diseñaron explícitamente para estas comparaciones.

**Conclusión:**

La elección de la estrategia de muestreo puede afectar de manera significativa a los resultados del xenomonitoreo molecular. Se requieren más investigaciones para determinar la estrategia óptima teniendo en cuenta las limitaciones logísticas y los contextos epidemiológicos.

## ملخص

**الغرض:**

استكشاف تأثير كل من طرق جمع البعوض، وكثافة جمع العينات، والجنس المستهدف، على الكشف بالرصد الخارجي الجزيئي عن الطفيليات المسببة لداء الفيلاريات اللمفي.

**الطريقة:**

قمنا بالبحث بشكل منهجي في خمس قواعد بيانات للدراسات، التي استخدمت استراتيجيتين أو أكثر لجمع عينات من البعوض البري، وقمنا بالاستعانة بطرق جزيئية لتقييم مدى انتشار الرصد الخارجي الجزيئي للطفيليات المسؤولة عن داء الفيلاريات اللمفي. قمنا بإجراء التحليلات التلوية العامة للتباين العكسي، واستكشفنا مصادر عدم التجانس باستخدام تحليلات المجموعات الفرعية. قمنا بتقييم الجودة المنهجية ومدى التيقن من الأدلة.

**النتائج:**

قمنا بتحديد 25 دراسة مؤهلة، حيث تم تحليل عدد 172083 بعوضة. لاحظنا ارتفاعًا ملموسًا في معدل انتشار الرصد الخارجي الجزيئي باستخدام طرق الجمع التي تستهدف البعوض الذي يتغذى على الدم، مقارنة بالطرق التي تستهدف البعوض غير المتغذى (بنسبة أرجحية مقدارها: 3.53؛ بفاصل ثقة مقداره %95: 1.52 إلى 8.24)، ولكن لا يوجد فرق ملموس مقارنة بطرق الجمع المحملة (بنسبة أرجحية مقدارها: 1.54؛ ‏بفاصل ثقة مقداره %95: 0.46 إلى 5.16). فيما يتعلق بالجنس، لاحظنا ارتفاعًا ملموسًا في معدل انتشار الرصد الخارجي الجزيئي لبعوض الأنوفيلين، مقارنة ببعوض الكوليسين في المناطق التي تكون فيها فصائل الأنوفيلة هي الناقل الرئيسي (بنسبة أرجحية مقدارها: 6.91؛ ‏بفاصل ثقة مقداره %95: 1.73 إلى 27.52). قدمت إحدى الدراسات أدلة على أن تقليل عدد مواقع جمع العينات لم يؤثر بشكل كبير على معدل انتشار الرصد الخارجي الجزيئي. إن الأدلة على وجود اختلافات في انتشار الرصد الخارجي الجزيئي بين استراتيجيات جمع العينات، تم اعتبار أنها منخفضة المصداقية، ويرجع ذلك جزئيًا إلى القيود الراسخة في الدراسات الرصدية التي لم يتم تصميمها بشكل صريح لهذه المقارنات.

**الاستنتاج:**

يمكن أن يؤثر اختيار استراتيجية جمع العينات بشكل كبير على نتائج الرصد الخارجي الجزيئي. هناك حاجة إلى مزيد من البحث لتحديد أفضل استراتيجية في ضوء القيود اللوجستية والأوضاع الوبائية.

## 摘要

**目的:**

探索蚊虫采集方法、采集强度和目标属对导致淋巴丝虫病的寄生虫分子异种监测的影响。

**方法:**

我们系统地检索了 5 个数据库中使用两种或两种以上方法采集野生蚊子样本的研究，并采用分子方法评估导致淋巴丝虫病的寄生虫的分子异种监测的患病率。我们进行了一般逆方差荟萃分析，并使用亚组分析探索异质性的来源。我们评估了方法学质量和证据的确定性。

**结果:**

我们确定了 25 项符合条件的研究，分析了 172,083 只蚊子。我们观察到，与针对未喂食蚊子的方法相比，针对吸血蚊子的收集方法的分子异种监测患病率明显更高（患病率：3.53；95％ 置信区间，CI：1.52–8.24)，但与妊娠采集方法相比，其差异无统计学意义（患病率：1.54；95% CI：0.46-5.16)。在属方面，我们观察到疟蚊的分子异种监测的患病率明显高于在*按蚊*为主要媒介的地区的库蚊的异种监测的患病率（患病率：6.91；95% CI：1.73-27.52)。一项研究提供的证据表明，减少采样点的数量不会显著影响分子异种监测的患病率。不同采样策略之间分子异种监测的患病率差异的证据被认为是低确定性的，部分原因是观察性研究的固有局限性，这些研究没有明确设计用于进行这些比较。

**结论:**

采样策略的选择对分子异种监测结果有显著影响。需要进一步研究，以便根据后勤限制和流行病学背景为最佳策略提供信息。

## Резюме

**Цель:**

Изучить влияние методов отбора комаров, интенсивности отбора образцов и подбора родов-мишеней на обнаружение вызывающих лимфатический филяриоз паразитов методом молекулярного ксеномониторинга.

**Методы:**

Проведен систематический поиск по пяти базам данных для выявления исследований, в которых использовались две или более стратегии сбора образцов диких комаров и применялись молекулярные методы для оценки распространенности паразитов, вызывающих лимфатический филяриоз, методом молекулярного ксеномониторинга. Проведен общий метаанализ с обратной дисперсией, и изучены источники гетерогенности с помощью анализа подгрупп. Проведена оценка методологической достоверности и качества доказательств.

**Результаты:**

Выявлено 25 приемлемых исследований, в которых проанализировано 172 083 комара. Распространенность, по данным молекулярного ксеномониторинга, оказывается значительно выше при использовании методов сбора, направленных на уже насытившихся кровью комаров, по сравнению с методами, направленными на голодных комаров (коэффициент распространенности: 3,53; 95%-й ДИ: 1,52–8,24), но без существенной разницы по сравнению с методами сбора, направленными на беременных самок (коэффициент распространенности: 1,54; 95%-й ДИ: 0,46–5,16). Что касается рода, то, по данным молекулярного ксеномониторинга, наблюдалась значительно более высокая распространенность для малярийных комаров по сравнению с комарами-кулицинами в районах, где виды *Anopheles* являются основными переносчиками (коэффициент распространенности: 6,91; 95%-й ДИ: 1,73–27,52). Результаты одного из исследований свидетельствуют о том, что сокращение числа мест отбора образцов не оказало существенного влияния на распространенность, по данным молекулярного ксеномониторинга. Доказательства различий в распространенности, по данным молекулярного ксеномониторинга, при разных стратегиях отбора образцов были сочтены малоубедительными, что отчасти объясняется присущими исследованиям методом наблюдения ограничениями, которые не были предназначены непосредственно для таких сравнений.

**Вывод:**

Выбор стратегии отбора образцов может оказать существенное влияние на результаты молекулярного ксеномониторинга. Для определения оптимальной стратегии с учетом логистики в условиях ограничений и эпидемиологических условий требуется проведение дальнейших исследований.

## Introduction

Lymphatic filariasis is a disabling and debilitating disease caused by the filarial worms *Wuchereria bancrofti, Brugia malayi* or *B. timori* that are transmitted by mosquitoes of the genera *Culex, Anopheles, Aedes* and *Mansonia*. The disease is targeted for elimination using mass drug administration and vector control; many countries have already achieved the goal of elimination as a public health problem. The World Health Organization (WHO) recommends that countries continue disease surveillance using cross-sectional surveys, routine surveillance of target populations, or molecular xenomonitoring to ensure infection levels remain below target thresholds or to confirm interruption of transmission.^1^

Molecular xenomonitoring is used as a surveillance strategy for vector-borne diseases such as lymphatic filariasis and onchocerciasis. The technique detects the presence of pathogen genetic material such as deoxyribonucleic acid (DNA) in disease vectors (e.g. mosquitoes). This method therefore gives a measure of the vector population’s exposure to pathogens picked up from infected humans, allowing it to serve as a proxy for the presence of human disease.

Molecular xenomonitoring overcomes many of the key challenges of lymphatic filariasis case surveillance: it does not rely on human blood sampling, it is relatively inexpensive and it allows integrated surveillance of multiple diseases.^2^ Innovations in mosquito trap design and field-friendly amplification and detection techniques are now bringing molecular xenomonitoring into the reach of control programmes, even those that lack specialist entomology training.^3^ A meta-analysis showed that molecular xenomonitoring had a high sensitivity at low microfilaria prevalence in communities, and demonstrated a strong correlation between molecular xenomonitoring and microfilaria prevalence when a consistent method is applied.^4^

A major limitation of molecular xenomonitoring is that there is no standardized protocol for sampling mosquitoes.^3^ Current WHO guidelines on molecular xenomonitoring^5^ indicate that any collection method can be used, and provide minimal instruction on frequency, scale, target species and sample sizes. However, each of these variables may influence the likelihood of a molecular xenomonitoring survey detecting mosquitoes positive for filarial DNA. Consequently, standardized guidelines, along with those tailored to specific settings, are needed. These guidelines will ensure that collection strategies effectively detect areas of potential disease transmission, and that results are comparable across timepoints and evaluation units. Developing such guidelines for sampling mosquitoes requires an understanding of how collection strategies influence the prevalence of filarial DNA in wild-caught mosquitoes.

Several mosquito collection methods can be used for lymphatic filariasis xenomonitoring.^5^ Each method exploits a specific stage of the mosquito’s gonotrophic cycle and therefore predominantly collects, though not exclusively, mosquitoes from that stage. In this review we have broadly categorized these methods into three groups: (i) fed collection methods, which target mosquitoes that have recently fed on blood. These methods use indoor resting catches (in which mosquitoes are collected either by direct aspiration or through the use of insecticides), or exit traps (traps fixed to windows to collect mosquitoes as they attempt to leave a building); (ii) gravid collection methods, which lure and trap gravid, ovipositing females using gravid traps; and (iii) unfed collection methods, which trap mosquitoes by exploiting their host-seeking behaviour. These methods use light traps (with or without carbon dioxide), odour-baited traps (such as the Biogents’ BG-Sentinel trap, Regensburg, Germany) or human landing catches (in which mosquitoes are caught as they alight on a human collector).

As the presence of parasite DNA in the mosquito is dependent on a previous bloodmeal from a lymphatic filariasis-infected host, bloodfed or gravid mosquitoes have a higher likelihood of containing filarial DNA than unfed mosquitoes.

A second factor that may affect the molecular xenomonitoring prevalence is the intensity of the sampling protocol. Lymphatic filariasis is a highly focal disease,^6^ and local transmission is influenced by a combination of environmental, climatic and socioeconomic conditions. Small hotspots of high transmission can persist in districts that have very low transmission overall.^7^^,^^8^ The likelihood of detecting a positive mosquito may increase with a higher number of sampling locations.

A third factor affecting molecular xenomonitoring prevalence is the predominant genus of mosquitoes collected. Molecular xenomonitoring prevalence is likely to be higher for mosquitoes that act as vectors for the disease than those that do not. In competent vectors, microfilariae that have been ingested develop into infective stage larvae, a process that takes a minimum of 10–12 days, so parasite DNA may be detectable for the remainder of the mosquito’s life.^9^^,^^10^ In non-competent vectors, parasite DNA is transient and often expelled within 48 hours of bloodmeal ingestion.^11^ Another consideration is bloodmeal size; *Culex quinquefasciatus* can ingest twice as man*y* microfilariae as *Aedes aegypti* under experimental conditions.^12^ Anthropophilic (human-seeking) vectors, such as *Anopheles gambiae*, are also likely to have greater exposure to filarial DNA than those that feed from a variety of hosts.

In this systematic review we aimed to determine how sampling strategy affects molecular xenomonitoring prevalence and informs molecular xenomonitoring implementation. We compared molecular xenomonitoring prevalence when measured using two or more different methods from within the following three categories: (i) mosquito collection methods: fed, gravid and unfed; (ii) sampling intensity; and (iii) mosquito genera: *Anopheles*, *Culex*, *Mansonia*, *Aedes* and *Armigeres*.

## Methods

We followed the Preferred Reporting Items for Systematic Reviews and Meta-Analyses guidelines^13^ using pre-determined methods from a protocol registered with the PROSPERO international database of prospectively registered systematic reviews (registration number CRD42020200351).^14^

### Eligibility criteria

Studies were included in our review if they met all of the following criteria: (i) they used two or more collection strategies; (ii) wild mosquito populations were sampled; and (iii) they used molecular methods (polymerase chain reaction (PCR), genetic sequencing or loop-mediated isothermal amplification) to both measure and report the molecular xenomonitoring prevalence for the causative agents of lymphatic filariasis (*W. bancrofti, B.malayi, B. timori*). We did not apply any language restrictions to the inclusion criteria.

### Search

We searched five bibliographic databases (CINAHL Complete, eBook Collection, Global Health, Global Health Archive and MEDLINE Complete) for all records up to and including 8 February 2023 using the EBSCO*host* research platform.^15^

We also checked the reference lists in included studies to identify further studies meeting the inclusion criteria. The search strategy is outlined in Box 1. Two reviewers assessed abstracts and selected papers for full-text screening. We resolved discrepancies by thorough review and discussion.

Box 1 **Search terms used in the systematic review on the impact of mosquito sampling strategies on molecular xenomonitoring prevalence for filariasis**

((Xenosurveillance OR Xeno-surveillance) OR (Xenomonitor* OR Xeno-monitor*) OR

((“Molecular screen*” OR “Molecular diagnos*” OR PCR OR “Polymerase chain reaction” OR sequencing OR LAMP)) AND (Mosquito* OR *Aedes* OR *Anopheles* OR *Culex* OR Mansonia))) AND (Onchocerc* OR “River blindness” OR Filaria* OR Elephantiasis OR “Wucheria bancrofti” OR “W.*°*bancrofti” OR “Brugia malayi” OR “B.*°*malayi” OR “Brugia timori” OR “B.*°*timori” OR Loa OR Loiasis OR “African eye worm” OR Mansonel*

Note: This search strategy was used for another research project which identified molecular xenomonitoring studies for any disease caused by filarial worms.^4^^,^^16^ Specific inclusion criteria for this review restricted studies to those relating only to lymphatic filariasis.

### Data extraction

We extracted study information at the smallest available level, e.g. individual villages within a district. Where molecular xenomonitoring prevalence and associated 95% confidence intervals (CIs) were not reported, we calculated these using data reported in the study. Where mosquitoes were screened in pools, we estimated the molecular xenomonitoring prevalence and 95% CI from the total number of mosquitoes screened, the size of the pools and the proportion of pools that were positive using the Poolscreen algorithm, version 2.0.^17^ If the sizes of individual pools were not reported, we used the mean pool size for Poolscreen calculations.

### Methodological quality assessment

We used the QUADAS–2 tool to assess the methodological quality of the included studies.^18^ The tool (available in the online repository)^19^ was adapted for evaluation of community-level surveillance methods before the start of the screening process.

### Statistical analysis and data synthesis

We calculated prevalence ratios with 95% CIs using RevMan 5 (Cochrane, London, United Kingdom of Great Britain and Northern Ireland) to compare molecular xenomonitoring prevalence between different sampling strategies. Because pooled mosquito data have a higher level of uncertainty than the same number of samples screened individually, we transformed the reported samples sizes to effective samples sizes (available in the online repository)^19^ to ensure RevMan calculated the correct CIs.

We conducted all meta-analyses using generic inverse variance models in RevMan5. We performed fixed effects meta-analyses if heterogeneity was absent or moderate (*I*^2^ < 60%), and random effects meta-analyses if heterogeneity was considerable or substantial (*I*^2^ ≥ 60%).^20^

We performed subgroup analyses by filarial parasite species, primary vector genus and trapping methods used to explore reasons for substantial heterogeneity. To assess significant differences between subgroups, we used *χ*^2^-squared tests with *P*-values less than 0.1 deemed statistically significant. We also performed sensitivity analyses to evaluate the effect of exclusion of trials that had a high risk of bias for any of the QUADAS-2 domains. Where 10 or more studies were included in a meta-analysis, we investigated the risk of publication bias using funnel plots.^21^

We assessed the certainty that the true differences between sampling methods lie close to those estimated by our meta-analyses using the Grading of Recommendations Assessment, Development and Evaluation approach.^22^ As all the included studies were observational studies, the evidence for each outcome started as low certainty; this grade could be further downgraded due to concerns about any of the following five domains: risk of bias; imprecision; inconsistency; indirectness; and reporting bias.^23^

## Results

**Fig. 1:**
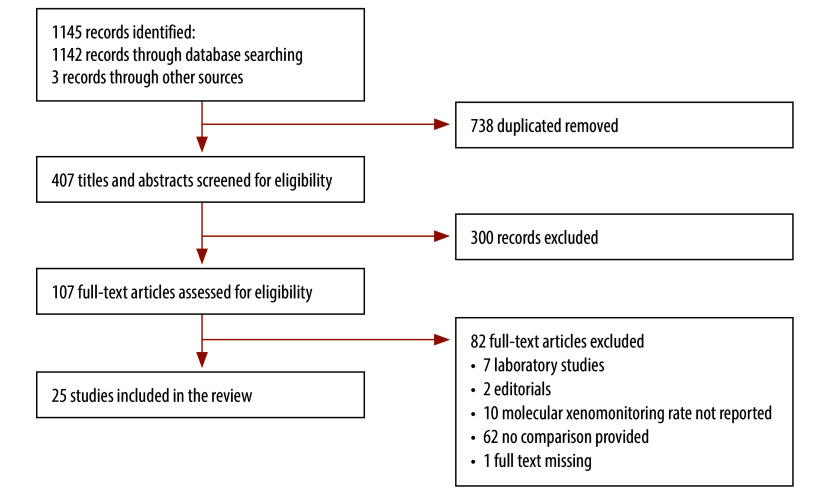
Flowchart showing the selection of studies included in the systematic review on the impact of mosquito sampling strategies on molecular xenomonitoring prevalence for filariasis

We identified 1142 records through electronic database searching, and three records through contact with study authors. After removal of duplicates, we screened 407 records. Of these, we identified 107 records for full-text assessment and 25 of these met the inclusion criteria.^3^^,^^24^^–^^47^ These were included in qualitative and quantitative synthesis (Fig. 1).

### Characteristics of included studies

Of the included studies, 12 studies were conducted in the WHO African Region, eight in the South-East Asia Region, two each in the Western Pacific Region and in the Region of the Americas, and one in the Eastern Mediterranean Region. The study characteristics are summarized in Table 1. A total of 172 083 mosquitoes were analysed across the included studies with a mean of 6373 per study (range: 208–57 357).

**Table 1:** Characteristics of included studies in the systematic review on the impact of mosquito sampling strategies on molecular xenomonitoring prevalence for filariasis

Study	Study location (country or territory)	Setting	Parasite of interest	Primary vector	No. of mosquitoes	Comparison
**Comparison 1: collection methods**						
Bockarie et al., 2000^24^	Papua New Guinea	Rural	*W. bancrofti*	*An. punctulatus*	621	Human landing catch versus light trap
Coulibaly et al., 2022^25^	Mali	Rural	*W. bancrofti*	*An. gambiae*	1364	Human landing catch versus Ifakara tent trap versus Biogents’ sentinel trap
Hoti et al., 2002^26^	India	Rural	*W. bancrofti*	*Cx. quinquefasciatus*	4940	Human landing catch versus indoor resting catch
Irish et al., 2015^27^	United Republic of Tanzania	Rural	*W. bancrofti*	*Cx. quinquefasciatus*	5737	Gravid trap versus light trap
Njenga et al., 2022^28^	Kenya	Rural	*W. bancrofti*	*An. gambiae s.l.* and *Cx. quinquefasciatus*	3652	Ifakara tent trap versus light trap
Opoku et al., 2018^29^	Ghana	Rural	*W. bancrofti*	*An. gambiae s.l.* and *An. funestus*	734	Gravid trap versus light trap versus Biogents’ sentinel trap versus exit trap versus indoor resting catch
Owusu et al., 2015^30^	Ghana	Rural	*W. bancrofti*	*An. gambiae s.l.*	4500	Gravid trap versus indoor resting catch
Pam et al., 2017^31^	Nigeria	Urban	*W. bancrofti*	*An. gambiae s.l.*	10 528	Gravid trap versus exit trap versus indoor resting catch
Pryce et al., 2022^32^	Cameroon	Rural	*W. bancrofti*	*An. gambia s.l.*	376	Indoor resting catch versus Biogents’ sentinel trap
Ramesh et al., 2018^3^	Brazil	Urban	*W. bancrofti*	*Cx. quinquefasciatus*	856	Indoor resting catch versus light trap
**Comparison 2: sampling intensity**						
Rao et al., 2016^33^	Sri Lanka	Rural	*W. bancrofti*	*Cx. quinquefasciatus*	57 357	300 versus 150 versus 75 trap locations
**Comparison 3: mosquito genera**						
de Souza et al., 2014^34^	Sierra Leone and Liberia	Urban	*W. bancrofti*	*An. gambiae s.l.*	16 073	*Anopheles* versus *Culex*
Dyab et al., 2016^35^	Egypt	Both	*W. bancrofti*	*Cx. pipiens*	1600	*Aedes* versus *Anopheles* versus *Culex*
Entonu et al., 2020^36^	Nigeria	Rural	*W. bancrofti*	*An. gambiae*	3000	*Anopheles* versus *Culex* versus *Aedes*
Fischer et al., 2002^37^	Indonesia	Rural	*B. timori*	*An. barbirostris*	1266	*Anopheles* versus *Culex*
Kouassi et al., 2015^38^	Guinea	Urban	*W. bancrofti*	*An. gambiae s.l.*	3747	*Anopheles* versus *Culex*
Lupenza et al., 2021^39^	United Republic of Tanzania	Rural	*W. bancrofti*	*Cx. quinquefasciatus*	7346	*Culex* versus *Anopheles*
McPherson et al., 2022^40^	Samoa	Rural	*W. bancrofti*	*Ae. polynesiensis*	8506	*Aedes* versus *Culex*
Mulyaningsih et al., 2019^41^	Indonesia	Rural	*B. malayi*	Unknown. Typically *Mansonia spp.*	1280	*Armigeres* versus *Culex* versus *Mansonia*
Nirwan et al., 2022^42^	Indonesia	Rural	*W. bancrofti, B. malayi, B. timori*	Unknown in this location	3907	*Armigeres* versus *Culex* versus *Mansonia* versus *Aedes*
Njenga et al., 2022^28^	Kenya	Rural	*W. bancrofti*	*An. gambiae s.l.* and *Cx. quinquefasciatus*	3652	*Anopheles* versus *Culex* versus *Mansonia* vs *Aedes*
Nurjana et al., 2020^43^	Indonesia	Rural	*B. malayi*	Unknown in this location	2989	*Culex* versus *Mansonia* versus *Armigeres* versus *Aedes* versus *Anopheles*
Pryce et al., 2022^32^	Cameroon	Rural	*W. bancrofti*	*An. gambiae s.l.*	350	*Anopheles* versus *Culex*
Ridha et al., 2020^44^	Indonesia	Rural	*W. bancrofti* and *B. malayi*	Unknown in this location	802	*Anopheles* versus *Culex* versus *Mansonia*
Schmaedick et al., 2014^45^	American Samoa	Rural	*W. bancrofti*	*Ae. polynesiensis*	17 448	*Aedes* versus *Culex*
Supriyono & Tan, 2020^46^	Indonesia	Rural	*B. malayi*	Unknown. Typically *Mansonia spp.*	208	*Aedes* versus *Anopheles* versus *Culex* versus *Mansonia*
Yokoly et al., 2020^47^	Côte d'Ivoire	Rural	*W. bancrofti*	*An. gambiae s.l.*	9244	*Anopheles* versus *Culex*

*Ae.*: *Aedes*; *An.: Anopheles*; *B.*: Brugia; *Cx.*: *Culex*; *s.l.*: sensu lato; *spp*.: species.

Note: Biogents AG is located in Regensburg, Germany.

### Methodological quality

We provide a summary of the methodological quality in Table 2. The sampling methods used in two studies were judged to introduce a high risk of bias.^26^^,^^39^ One study^26^ screened mosquitoes caught by the indoor resting catch method in pools of 10, and those caught by human landing catch method in pools of 50; their study also reported reduced sensitivity of molecular detection in pool sizes of 50 compared to microscopic detection following dissection. This approach prevents an unbiased comparison of mosquitoes collected by the two catch methods. Another study^39^ used light traps to preferentially collect *Anopheles* mosquitoes and gravid traps to preferentially collect *Culex* mosquitoes, and they presented their data according to species. The comparisons we have made between species are likely confounded by the fact that gravid traps collect mosquitoes that have taken at least one bloodmeal.

**Table 2:** Methodological quality assessment of studies included in the systematic review on the impact of mosquito sampling strategies on molecular xenomonitoring prevalence for filariasis

	Study	Sampling site selection risks	Sampling site selection applicability	Sampling methods risk	Sampling methods applicability	Flow and timing risks
**Comparison 1: collection methods**					
	Bockarie et al., 2000^24^	Low	Low	Unclear	Low	Low
	Coulibaly et al., 2022^25^	Low	Low	Low	Low	Low
	Hoti et al., 2002^26^	Unclear	Low	High	Low	Unclear
	Irish et al., 2015^27^	Low	Low	Low	Low	Low
	Njenga et al., 2022^28^	Low	Low	Low	Low	Low
	Opoku et al., 2018^29^	Unclear	Low	Low	Low	Low
	Owusu et al., 2015^30^	Low	Low	Unclear	Low	Unclear
	Pam et al., 2017^31^	Low	Low	Unclear	Low	Unclear
	Pryce et al., 2022^32^	Low	Unclear	Unclear	Low	Low
	Ramesh et al., 2018^3^	Low	Low	Low	Low	Low
**Comparison 2: sampling intensity**					
	Rao et al., 2016^33^	Low	Low	Low	Low	Low
**Comparison 3: mosquito genera**					
	de Souza et al., 2014^34^	Low	Low	Low	Low	Low
	Dyab et al., 2016^35^	Unclear	Low	Low	Low	Low
	Entonu et al., 2020^36^	Low	Low	Low	Low	Low
	Fischer et al., 2002^37^	Unclear	Low	Low	Low	Low
	Kouassi et al., 2015^38^	Low	Low	Unclear	Low	Low
	Lupenza et al., 2021^39^	Low	Low	High	Low	Low
	McPherson et al., 2022^40^	Low	Low	Low	Low	Low
	Mulyaningsih et al., 2019^41^	Unclear	Low	Low	Low	Low
	Nirwan et al., 2022^42^	Low	Low	Low	Unclear	Low
	Njenga et al., 2022^28^	Low	Low	Low	Low	Low
	Nurjana et al., 2020^43^	Low	Low	Low	Low	Low
	Pryce et al., 2022^32^	Low	Unclear	Unclear	Low	Low
	Ridha et al., 2020^44^	Unclear	Low	Low	Unclear	Low
	Schmaedick et al., 2014^45^	Low	Low	Low	Low	Low
	Supriyono & Tan, 2020^46^	Unclear	Low	Low	Low	Low
	Yokoly et al., 2020^47^	Unclear	Low	Unclear	Low	Low

Note: Some articles are included in more than one comparison and hence appear twice in the table.

### Summary of findings

Molecular xenomonitoring prevalence was significantly higher when fed collection methods were used compared with unfed. Higher prevalence was also observed in anopheline mosquitoes than culicine mosquitoes. However, the certainty of the evidence scored very low (Table 3). A summary of the assessments and the justifications for each rating are also provided in Table 3. Below, we discuss in detail the data underpinning each of these assessments.

**Table 3:** The relative effect of different mosquito sampling strategies on molecular xenomonitoring prevalence and evaluations of the certainty of the evidence

Comparison	Relative effect, prevalence ratio (95% CI)	No. of mosquitoes (studies)	Quality of the evidence	Comments
**Comparison 1: collection methods**				
Fed versus gravid collection methods	1.54 (0.46–5.16)	12 711 (3)	Very low^a,b,c^	Downgraded for imprecision, inconsistency and indirectness
Fed versus unfed collection methods	3.53 (1.52–8.24)	5 167 (2)	Very low^d,e^	Downgraded for indirectness and risk of bias
Gravid versus unfed collection methods	0.20 (0.01–3.40)	5 927 (1)	Very low^f,g^	Downgraded for serious imprecision and indirectness
**Comparison 2: sampling intensity**				
300 versus 150 trapping locations	0.89 (0.54–1.46)	29 797 (1)	Very low^g^	Downgraded for indirectness
300 versus 75 trapping locations	0.90 (0.54–1.51)	28 624 (1)	Very low^g^	Downgraded for indirectness
150 versus 75 trapping locations	1.01 (0.60–1.68)	27 921 (1)	Very low^g^	Downgraded for indirectness
**Comparison 3: mosquito genera**				
*Anopheles* versus *Culex* mosquitoes:				
*Anopheles* areas	6.91 (1.73–27.52)	28 974 (6)	Very low^b,h^	Downgraded for inconsistency and imprecision
*Culex* areas	2.68 (0.08–94.93)	53 610 (2)	Very low^a^	Downgraded for imprecision
*Aedes* versus *Culex* mosquitoes	1.07 (0.52–2.19)	64 705 (2)	Very low^a,i^	Downgraded for imprecision and indirectness

CI: confidence interval.

^a^ Downgraded for imprecision: the 95% CIs include both no difference and a large difference.

^b^ Downgraded for inconsistency: there was substantial heterogeneity that could not be explained by subgroup analyses.

^c^ Downgraded for indirectness: all studies were conducted in areas where the primary filariasis vectors belonged to the *Anopheles gambiae* species complex; the findings may not apply to other transmission areas.

^d^ Downgraded for indirectness: over 92% of the weight of the meta-analysis came from a single study; the findings may not apply to other transmission areas.

^e^ Downgraded for risk of bias: the sampling methods for the main study contributing to the analysis were judged to have a high risk bias as mosquitoes collected using fed traps were screened in smaller pool sizes than those collected by gravid traps; this may favour higher molecular xenomonitoring prevalence for mosquitoes from fed collection methods.

^f^ Downgraded twice for serious imprecision: the 95% CIs include large differences in favour of each type of collection method.

^g^ Downgraded for indirectness: a single study contributed to the meta-analysis; the findings may therefore not apply to other transmission areas.

^h^ Downgraded for imprecision: although the 95% CIs are extremely wide and include both a small difference and a very large difference.

^i^ Downgraded for indirectness: studies were conducted in American Samoa and Samoa; the findings may not apply to other transmission areas.

Note: We used the Grading of Recommendations Assessment, Development and Evaluation approach for grading the quality of the evidence.^22^

### Comparison 1: collection methods

#### Fed versus gravid

**Fig. 2:**
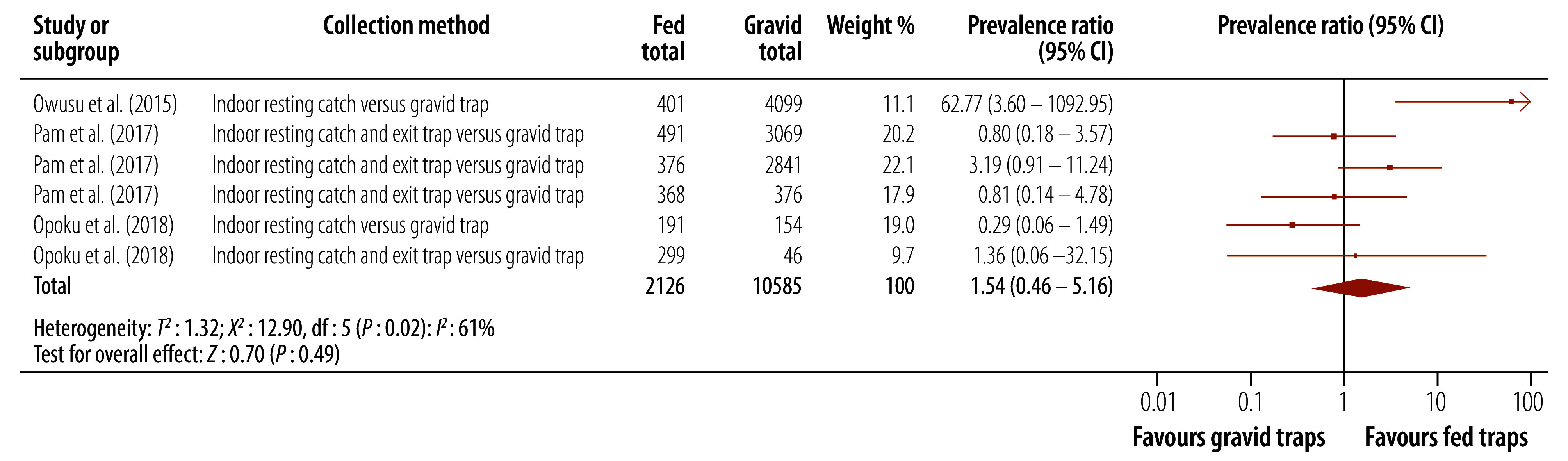
Effect of mosquito collection method, fed versus gravid, on molecular xenomonitoring prevalence

For this subgroup analysis, we included three studies.^29^^–^^31^ Higher molecular xenomonitoring prevalence was observed for mosquitoes collected using fed traps than for mosquitoes collected using gravid traps, but this difference was not statistically significant (prevalence ratio: 1.54; 95% CI: 0.46–5.16; Fig. 2). There was substantial heterogeneity between studies (*I*^2^: 61%). A subgroup analysis by the specific type of trapping method used did not explain this heterogeneity.

CI: confidence interval.

Note: We used a random-effects meta-analysis.

#### Fed versus unfed

**Fig. 3:**
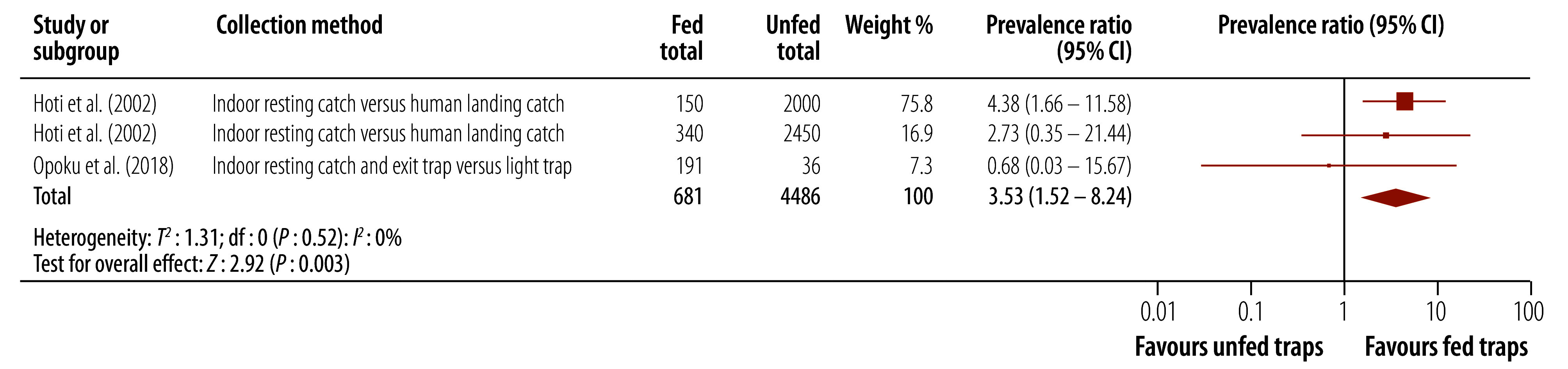
Effect of mosquito collection method, fed versus unfed, on molecular xenomonitoring prevalence

Analysis from two studies showed that molecular xenomonitoring prevalence wasapproximately 3.5 times higher for mosquitoes collected using fed traps than for mosquitoes collected using unfed traps (prevalence ratio: 3.53; 95% CI: 1.52–8.24; Fig. 3).^26^^,^^29^ Note that 92.7% of the weight of the meta-analysis came from a single study^26^ that was considered at high risk of bias. A sensitivity analysis excluding this study suggested there was no difference between the two collection methods.

CI: confidence interval.

Note: We used a fixed-effects meta-analysis.

#### Gravid versus unfed

Only one site provided evidence from sufficiently large effective sample sizes to contribute to the meta-analysis.^27^ This reported a higher molecular xenomonitoring prevalence for mosquitoes collected using unfed collection methods than for mosquitoes collected using gravid collection methods; however, this finding was not statistically significant (prevalence ratio: 0.20; 95% CI: 0.01–3.40).

### Comparison 2: sampling intensity

None of the included studies provided a comparison of different longitudinal collection intensities (for example, nightly collections versus monthly collections). One study compared molecular xenomonitoring prevalence based on different densities of trapping locations (300 versus 150 versus 75 locations) on *W. bancrofti* detection rates.^33^ The study found that sampling mosquitoes from 300 locations did not lead to higher molecular xenomonitoring prevalence than when sampling the same number of mosquitoes from 75 sampling locations (prevalence ratio: 0.90; 95% CI: 0.54–1.51). We did not observe any difference in molecular xenomonitoring prevalence between any of the three sampling strategies.

### Comparison 3: mosquito genera

#### Anopheles versus Culex

Eight studies, from 11 study sites, provided comparisons of molecular xenomonitoring prevalence for *Anopheles* and *Culex* mosquitoes.^30^^,^^31^^,^^34^^,^^35^^,^^37^^–^^39^^,^^47^

**Fig. 4:**
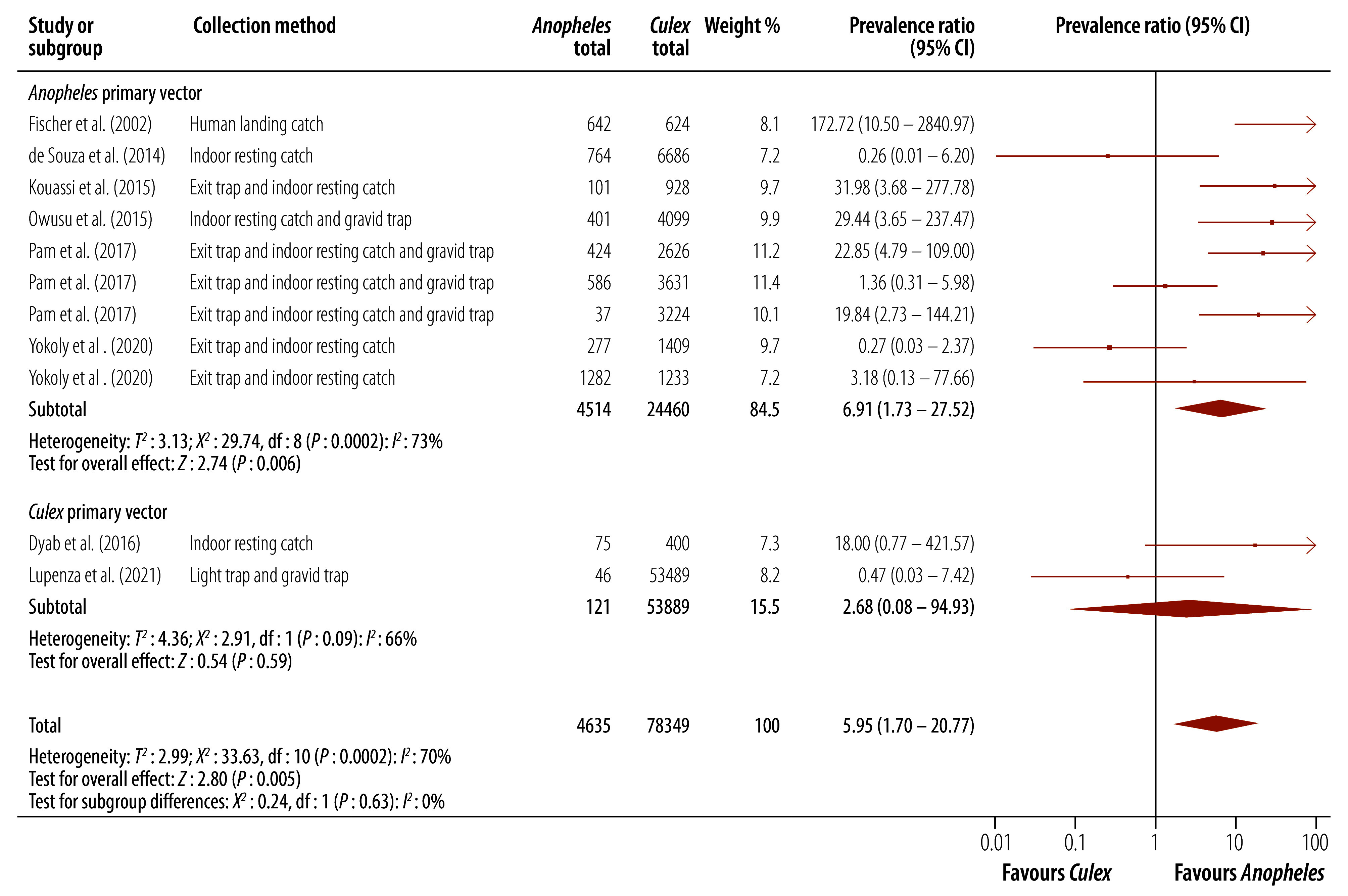
Effect of collected mosquito genera on molecular xenomonitoring prevalence

In almost all included studies, the numbers of *Culex* mosquitoes collected far outweighed the number of *Anopheles* mosquitoes. In areas where the primary vector is *Anopheles*, molecular xenomonitoring prevalence was approximately seven times higher for *Anopheles* mosquitoes than for *Culex* mosquitoes (prevalence ratio: 6.91; 95% CI: 1.73–27.52; Fig. 4). In areas of *Culex*-transmitted lymphatic filariasis, the molecular xenomonitoring prevalence was also higher for *Anopheles* mosquitoes, although the CI for this estimate was wide (prevalence ratio: 2.68; 95% CI: 0.08–94.93). In *Anopheles* areas, there was substantial heterogeneity between studies (*I*^2^: 73%) which was not explained by subgroup analyses. The high heterogeneity was due to several studies showing very large differences between mosquito genera. One study in Indonesia reported a much higher prevalence of *B. timori* DNA in *Anopheles* mosquitoes than *Culex* mosquitoes, resulting in a prevalence ratio of 172.7.^37^ Three additional studies also provided study areas with a prevalence ratio of 20 or greater.^30^^,^^31^^,^^38^

CI: confidence interval.

Note: We used a random-effects meta-analysis.

#### Aedes versus Culex

Two studies from American Samoa and Samoa, comprising 11 study sites, provided comparisons for a meta-analysis of molecular xenomonitoring prevalence between *Aedes* and *Culex* mosquitoes.^40^^,^^45^ There was no difference between the two genera (prevalence ratio: 1.07; 95% CI: 0.52–2.19).

#### Other mosquito genera

Six studies provided comparisons between other mosquito genera including *Armigeres* and *Mansonia* species.^28^^,^^41^^–^^44^^,^^46^ The limited number of studies contributing to each comparison and the number of mosquitoes positive for filarial DNA in each study precluded a quantitative synthesis for this outcome.

## Discussion

Our findings suggest that mosquito collection methods can have an important impact on molecular xenomonitoring prevalence, although precise estimates of the impact were difficult to obtain.

Multiple studies conducted in *Anopheles*-transmitted lymphatic filariasis areas reported substantially higher molecular xenomonitoring prevalence when targeting bloodfed mosquitoes than gravid mosquitoes. However, this effect was not consistent between studies and we were unable to determine the reasons for this heterogeneity. The lack of studies from areas where the primary vector is *Culex* is an important gap. Gravid traps are an efficient tool for collecting *Culex* mosquitoes, and have been used as the sole collection method for molecular xenomonitoring by elimination programmes in Bangladesh and Sri Lanka.^2^^,^^33^

Our meta-analysis showed a large difference in molecular xenomonitoring prevalence between fed and unfed collection methods. However, most of the weight of this analysis was contributed by a single study in an area of *Cx. quinquefasciatus-*transmitted lymphatic filariasis,^26^ and the applicability of this result to other transmission settings is limited. While it is logical that molecular xenomonitoring prevalence would be higher when targeting recently bloodfed mosquitoes, there remains uncertainty from the available data that this effect will always be observed.

Strong evidence was reported by one high quality study in Sri Lanka that a reduced number of sampling sites per evaluation unit, from 300 to 75, did not lead to reduced detection of *W. bancrofti* DNA in *Cx. quinquefasciatus*. This finding, observed in two post-transmission assessment survey communities, supports the feasibility of molecular xenomonitoring, although evidence from other areas will be required to determine whether this approach is applicable to other transmission zones.

Lymphatic filariasis programmes looking for the most sensitive approach for detecting *Anopheles*-transmitted lymphatic filariasis may wish to consider using a sampling strategy that preferentially targets *Anopheles* mosquitoes, since these methods are likely to collect a higher proportion of parasite-positive mosquitoes. However, this advantage will need to be balanced against the convenience of other collection methods such as gravid traps (which are an efficient method for collecting large numbers of *Culex* mosquitoes).^9^ Historically, the collection of bloodfed *Anopheles* mosquitoes has depended on indoor collections using aspirators or pyrethrum spray – an approach that is labour-intensive and typically results in modest collection numbers.^2^ Given the significantly higher molecular xenomonitoring prevalence for *Anopheles* mosquitoes, programmatic use of molecular xenomonitoring for the detection of ongoing cases of lymphatic filariasis in extremely low prevalence areas may depend upon the development of new tools that are efficient at collecting bloodfed or gravid *Anopheles* mosquitoes.

We assume that the primary explanation for variation in sensitivity between studies is the differences in the sampling strategy. However, primer and probe design, as well as the equipment used, can affect PCR results, and direct comparisons between molecular xenomonitoring methods have shown variation in sensitivity.^48^^,^^49^

There are narrative reviews on the status of molecular xenomonitoring. For example, one review^50^ highlighted the need for systematic methods and new WHO guidelines to be developed to supplement post-validation surveillance. Another review^51^ proposed that molecular xenomonitoring has enormous potential for the surveillance of vector-borne diseases, with the capacity for it to replace (rather than supplement) current human surveillance strategies. However, they identified several key barriers that must be overcome, including the development of protocols that account for heterogeneity in pathogen infection rates both within the mosquito and the human population.

Our review provides evidence supporting the development of standardized molecular xenomonitoring sampling protocols that specifically consider mosquito collection methods and genus. However, the certainty of evidence for every comparison is very low due to the inherent limitations of observational data, and specific concerns regarding the comparisons drawn from the available literature. Consequently, there is a need for further research in these areas to inform an optimum molecular xenomonitoring sampling strategy.
